# Metabolomic Alterations Associated with Adjunctive Hydrogen Gas Inhalation During Concurrent Chemoradiotherapy in Locally Advanced Head and Neck Cancer: A Pilot Study

**DOI:** 10.3390/cancers18142191

**Published:** 2026-07-08

**Authors:** Imjai Chitapanarux, Narongchai Autsavapromporn, Wimrak Onchan, Somvilai Chakrabandhu, Pooriwat Muangwong, Apidet Duangya, Tanin Lertsiriladakul, Atikorn Panya, Atchara Paemanee

**Affiliations:** 1 Division of Radiation Oncology, Department of Radiology, Faculty of Medicine, Chiang Mai University, Chiang Mai 50200, Thailand; imjai.chitapanarux@cmu.ac.th (I.C.); wimrak.o@cmu.ac.th (W.O.); somvilai.chak@cmu.ac.th (S.C.); pooriwat.m@cmu.ac.th (P.M.); aphidet.d@cmu.ac.th (A.D.); tanin.l@cmu.ac.th (T.L.); 2 Food Biotechnology Research Team, Functional Ingredients and Food Innovation Research Group, National Center for Genetic Engineering and Biotechnology, National Science and Technology Development Agency, Khlong Luang, Pathum Thani 12120, Thailand; atikorn.pan@biotec.or.th (A.P.); atchara.pae@biotec.or.th (A.P.)

**Keywords:** metabolomics, locally advanced head and neck cancer, concurrent chemoradiotherapy, hydrogen gas, oxidative stress, purine metabolism, uric acid

## Abstract

**Background**: H_2_ gas inhalation has been proposed as a selective modulator of reactive oxygen species (ROS), potentially mitigating treatment-related oxidative damage. This study investigated the effects of adjunctive H_2_ gas inhalation on serum metabolomic profiles and clinical toxicities in patients with LAHNC undergoing CCRT. **Methods**: Twenty patients were prospectively randomized to receive either standard CCRT alone (Group A) or CCRT combined with adjunctive H_2_ gas inhalation (Group B). Serum samples collected before and after treatment were analyzed using untargeted ultra-high-performance liquid chromatography coupled with ion mobility quadrupole time-of-flight high-resolution mass spectrometry (UHPLC-IM-QTOF-HRMS)-based metabolomics. **Results**: One patient in Group B discontinued participation, leaving 19 patients for the final analysis. Patients receiving adjunctive H_2_ gas inhalation tended to exhibit numerically lower frequencies of moderate treatment-related toxicities, fewer chemotherapy delays, and a shorter overall treatment duration than those receiving CCRT alone. Metabolomic profiling in the CCRT-alone group revealed exploratory alterations in metabolites associated with arginine metabolism, glutathione metabolism, and purine metabolism following treatment. Ornithine, uric acid, and tetrahydrodeoxycorticosterone were among the candidate discriminative metabolites with exploratory discriminatory performance after CCRT. In contrast, patients receiving adjunctive H_2_ gas inhalation showed a more limited pattern of pathway mapping, with exploratory evidence from an illustrative single-hit pathway assignment suggesting possible involvement of purine metabolism. Altered uric acid levels together with changes in several lipid-related metabolites may collectively reflect metabolic responses associated with treatment-related oxidative stress during CCRT. Consistent with these findings, direct between-group comparison of within-subject changes (Δ = post − pre) indicated a numerically smaller reduction in serum uric acid levels in the adjunctive H_2_ gas inhalation group; however, this difference did not reach statistical significance. **Conclusions**: Adjunctive H_2_ gas inhalation during CCRT may be associated with reduced moderate treatment-related toxicities and exploratory changes in systemic metabolic profiles in patients with LAHNC. However, the study was not designed to evaluate oncological outcomes, and the metabolomic findings, particularly the pathway-level interpretations, should be considered exploratory because several pathway assignments were based on only one or a few mapped metabolites, together with the limited sample size, patient heterogeneity, and lack of independent external validation. Further validation in larger, independent cohorts using comprehensive between-group metabolomic analyses is warranted.

## Simple Summary

Previous studies investigating hydrogen (H_2_) gas inhalation in patients with locally advanced head and neck cancer (LAHNC) receiving concurrent chemoradiotherapy (CCRT) have suggested possible benefits in reducing treatment-related toxicities. However, the serum metabolomic profiles associated with the adjunctive H_2_ gas inhalation during CCRT remain poorly understood. Therefore, this study investigated serum metabolomic profiles in LAHNC patients receiving adjunctive H_2_ gas inhalation during CCRT. The findings revealed exploratory metabolic alterations involving metabolites associated with purine metabolism and lipid-related pathways, suggesting that H_2_ gas inhalation may be associated with partial modulation of systemic metabolic responses related to treatment-associated oxidative stress during CCRT.

## 1. Introduction

Concurrent chemoradiotherapy (CCRT) remains the standard treatment for patients with locally advanced head and neck cancer (LAHNC), providing superior tumor control and improved survival compared with radiation therapy (RT) alone. However, despite these clinical benefits, CCRT is frequently associated with substantial acute and chronic toxicities, including oral mucositis, dysphagia, xerostomia, and hearing impairment. These adverse effects can markedly impair quality of life and, in some cases, compromise treatment adherence and completion. Although advances in RT techniques, such as intensity-modulated radiotherapy (IMRT), have reduced certain complications, late toxicities remain clinically significant. A pooled analysis of 21 randomized controlled trials involving LAHNC patients treated with CCRT using modern RT techniques reported grade ≥ 3 late pharyngitis in 17%, grade ≥ 3 dysphagia in 16%, and grade ≥ 2 xerostomia in 38% of patients [1].

A central mechanism underlying radiation-induced tissue injury is the generation of reactive oxygen species (ROS), particularly hydroxyl radicals (^•^OH), which contribute to oxidative stress and cellular damage in normal tissues. Various strategies, including the use of free radical scavengers, have been explored to mitigate radiation-associated oxidative injury [2]. Among ROS, ^•^OH are highly reactive and cytotoxic, and their involvement in ionizing radiation-induced damage has been well established in both in vitro and in vivo models [3]. Although several conventional antioxidants have demonstrated radioprotective effects in preclinical settings [2], achieving selective protection of normal tissues without compromising tumor control remains a challenge.

Molecular hydrogen (H_2_) has recently emerged as a potential selective antioxidant. H_2_ has been proposed to preferentially interact with highly reactive oxidants, particularly hydroxyl radicals (^•^OH), while having minimal effects on physiologically important ROS involved in cellular signaling [3,4,5]. Preclinical studies have demonstrated that H_2_ reduces radiation-induced DNA damage and improves cell viability following RT [4,5]. Clinical investigations have further suggested that prolonged H_2_ gas inhalation is safe and well tolerated in healthy adults, supporting its potential therapeutic applicability [6]. Nevertheless, despite these encouraging findings, current evidence suggests that H_2_ is unlikely to function as a universal radioprotective agent, and its biological effects remain incompletely understood [3,4,5,6,7,8,9]. Therefore, additional mechanistic and clinical studies are needed to clarify its precise role during CCRT and to establish its clinical efficacy.

Consistent with these observations, several clinical studies have evaluated the safety and potential therapeutic benefits of H_2_ gas inhalation in patients with cancer. A retrospective study of 16 patients receiving IMRT showed that daily inhalation of 5% H_2_ for 30 min significantly attenuated radiation-induced bone marrow suppression, with higher white blood cell and platelet counts compared with controls, without apparent compromise of antitumor efficacy [7]. In another cohort study of 82 cancer patients, inhalation of H_2_ gas for ≥3 h daily over 3 months was associated with improvements in fatigue, insomnia, and pain [8]. Building on these findings, our previous pilot study demonstrated the feasibility and safety of daily H_2_ gas inhalation throughout 33 CCRT sessions in LAHNC patients, without inhalation-related adverse events, but did not investigate treatment-associated metabolic alterations [9].

Metabolomics has emerged as a powerful systems-level approach for investigating disease mechanisms, therapeutic responses, and biomarker discovery [10]. By profiling small-molecule metabolites in biological fluids, metabolomics provides insight into treatment-induced alterations in cellular and systemic biochemistry. In the present study, we aimed to characterize serum metabolomic changes in LAHNC patients receiving CCRT, with or without adjunctive H_2_ gas inhalation. To the best of our knowledge, this is one of the first studies to investigate serum metabolomic alterations associated with adjunctive H_2_ gas inhalation during CCRT in patients with LAHNC. We hypothesized that adjunctive H_2_ gas inhalation would be associated with exploratory alterations in metabolites related to oxidative stress and systemic metabolic responses during CCRT. Characterizing these metabolic changes may improve understanding of treatment-associated biological responses and generate hypotheses for future mechanistic and clinical studies of more tolerable and personalized therapeutic strategies for LAHNC. To complement the untargeted metabolomic analyses, direct between-group comparisons of within-subject treatment-associated changes (Δ = post − pre) were performed for selected metabolites to further explore the potential biological effects of adjunctive H_2_ gas inhalation.

## 2. Materials and Methods

### 2.1. Ethics

This prospective randomized interventional clinical trial was conducted to investigate the effects of adjunctive H_2_ gas inhalation on serum metabolomic changes in patients with LAHNC undergoing CCRT. The study was registered at the Thai Clinical Trials Registry (TCTR) under registration number TCTR20240515006 (registered on 15 May 2024). The study protocol was approved by the Human Research Ethics Committee of the Faculty of Medicine, Chiang Mai University, Thailand (approval date: 30 January 2024; study code: RAD-2566-0615; research ID: 615). The trial was conducted in accordance with the Declaration of Helsinki. Written informed consent was obtained from all participants prior to enrollment.

### 2.2. Study Subjects

**Figure 1:**
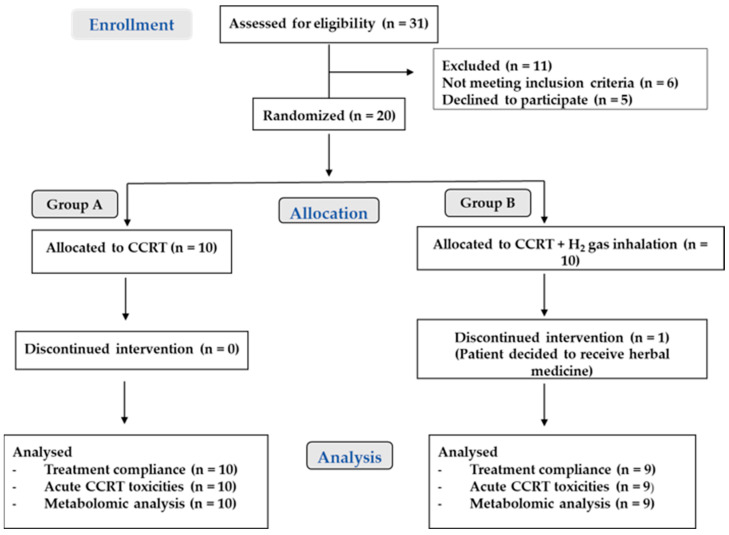
CONSORT flow diagram illustrating patient enrollment, allocation, follow-up, and analysis in this study. Twenty patients were randomized to receive concurrent chemoradiotherapy (CCRT) alone (*n* = 10) or CCRT combined with H_2_ gas inhalation (*n* = 10). One patient in the H_2_ inhalation group discontinued the intervention. Analyses included treatment compliance, acute CCRT-related toxicities, and metabolomic profiling.

Eligible participants were required to provide informed consent, be aged 18–70 years, have an Eastern Cooperative Oncology Group (ECOG) performance status of 0–2, and have a histologically confirmed diagnosis of locoregionally advanced head and neck cancer considered suitable for curative-intent CCRT. Exclusion criteria included distant metastasis, locoregional recurrence, prior head and neck irradiation, contraindications to RT, chemotherapy, or H_2_ gas inhalation, and the presence of a tracheostomy tube. Patients were enrolled between March 2024 and January 2025 (Figure 1).

### 2.3. H_2_ Gas Inhalation

Eligible patients were randomly assigned using a single-blind (assessor-blinded), block-randomized design, to either the control group (Group A), which received standard CCRT alone, or the experimental group (Group B), which received CCRT combined with daily H_2_ gas inhalation. Patients in Group B inhaled H_2_ gas for 1 h daily via nasal cannula, administered 1–2 h prior to each irradiation session. H_2_ gas was generated using a hydrogen generator (Hycellvator ET 100; Helix Japan Co., Ltd., Tokyo, Japan) at a flow rate of 1.67 L/min. Both groups received identical CCRT regimens, consisting of weekly cisplatin (40 mg/m^2^) or weekly carboplatin (area under the curve [AUC] = 2), depending on renal function, combined with IMRT delivered in 30–33 fractions over 6–6.5 weeks [9]. Patients in both groups were assessed weekly for CCRT-related toxicities according to the Common Terminology Criteria for Adverse Events (CTCAE), version 5.0 [11]. Treatment-related toxicities were assessed by physicians who were blinded to treatment allocation. The most severe adverse events experienced by each patient during the entire course of CCRT were recorded. Baseline patient characteristics are summarized in Table 1.

### 2.4. Sample Preparation

To minimize potential dietary interference, patients fasted for 10–12 h before blood collection. Peripheral venous blood samples were collected from all LAHNC patients immediately before initiation and immediately after completion of CCRT using serum-separating tubes. Samples were centrifuged at 3000 rpm for 10 min at room temperature, and the resulting serum was transferred into 1.5 mL microcentrifuge tubes and stored at −80 °C until analysis.

For metabolomic analysis, serum samples were thawed on ice, and 150 μL of serum was mixed with 450 μL of 70% methanol containing 55 mg/L 4-chloro-L-phenylalanine (Sigma-Aldrich, St. Louis, MO, USA) as an internal standard. The mixture was vortexed for 30 s and centrifuged at 13,000× *g* for 10 min at 4 °C. The supernatant was transferred to clean 1.5 mL tubes and filtered through a hydrophilized polytetrafluoroethylene (PTFE) membrane (0.22 μm pore size) prior to ultra-high-performance liquid chromatography coupled with ion mobility–quadrupole time-of-flight high-resolution mass spectrometry (UHPLC-IM-QTOF-HRMS; Agilent 6560, Agilent Technologies, Santa Clara, CA, USA) analysis.

### 2.5. Metabolomics Analysis

Untargeted metabolomic profiling was performed using an Agilent InfinityLab UHPLC system coupled with an Agilent 6560 IM-QTOF-MS system (Agilent Technologies, Santa Clara, CA, USA). Chromatographic separation was achieved using an Agilent InfinityLab Poroshell 120 HILIC-Z column (2.7 μm, 2.1 × 100 mm) equipped with a guard column. The column temperature was maintained at 30 °C with a flow rate of 0.3 mL/min. The mobile phases consisted of (A) 10 mM ammonium formate containing 0.1% (*v*/*v*) formic acid in water and (B) 10 mM ammonium formate containing 0.1% (*v*/*v*) formic acid in water/acetonitrile (1:9, *v*/*v*). The injection volume was 4 μL. The gradient elution program was as follows: 0–10 min, 100% to 70% B; 10–10.5 min, held at 70% B; 10.5–12 min, returned to 100% B; followed by re-equilibration at 100% B until 14 min. The total run time was 14 min.

Mass spectrometric detection was performed using electrospray ionization (ESI) in positive ion mode. Data were acquired in full-scan MS over an *m*/*z* range of 100–1700 using Auto MS/MS acquisition. Each sample was analyzed in triplicate (three technical replicate injections) to assess analytical reproducibility. In addition, pooled quality control (QC) samples, prepared by combining equal aliquots from all study samples, were injected periodically throughout the analytical sequence to monitor instrument stability and analytical performance. The source parameters were as follows: drying gas temperature, 225 °C; drying gas flow, 13 L/min; nebulizer pressure, 60 psi; sheath gas temperature, 340 °C; sheath gas flow, 12 L/min; and capillary voltage, 3000 V [12]. Because only positive-ion acquisition was performed, metabolites that preferentially ionize in negative mode may have been underrepresented. Accordingly, the present analysis provides partial metabolome coverage, and future studies incorporating both positive- and negative-ionization modes are warranted to achieve more comprehensive metabolite profiling.

### 2.6. Data Processing and Statistical Analysis

Raw LC-MS data were processed using Progenesis QI software (version 2.3; Nonlinear Dynamics, Newcastle, UK). The processed data were subsequently exported for downstream statistical analysis. A total of 1169 and 1162 metabolite features were initially detected in the pre- and post-CCRT and pre- and post-CCRT + H_2_ groups, respectively. After preprocessing and quality filtering, 385 and 389 metabolite features, respectively, were retained for further statistical analysis. Metabolite annotation was performed by matching metabolite features against the Human Metabolome Database (HMDB), the METLIN MS/MS spectral database, and the Siriraj Metabolomics Data Warehouse (SiMD) library using accurate precursor *m*/*z* values and MS/MS fragmentation spectra [13,14,15]. Matching criteria included a precursor mass tolerance of <5 ppm and a fragment mass tolerance of <5 ppm. Metabolite annotation confidence was assigned according to the Metabolomics Standards Initiative (MSI) guidelines. Because authentic reference standards were not analyzed, all reported metabolite identities were considered putatively annotated compounds. Collision cross section (CCS) information was not used for metabolite annotation. Exogenous compounds, drug-related metabolites, dietary-derived compounds, and unidentified metabolite features were excluded prior to downstream pathway enrichment and biological interpretation analyses to improve biological relevance and interpretability.

Processed datasets were analyzed using MetaboAnalyst 6.0. Continuous variables are presented as mean ± standard deviation (SD) or median, as appropriate. Data normality was assessed using the Shapiro–Wilk test. Paired and independent *t*-tests were used for within-group and between-group comparisons, respectively. When normality was not met, Wilcoxon signed-rank and Mann–Whitney U tests were applied. Clinical outcomes were summarized descriptively because of the pilot nature of the study and the limited sample size. Continuous clinical variables were compared using the Mann–Whitney U test. A two-sided *p* value < 0.05 was considered statistically significant.

Multivariate analyses were conducted using principal component analysis (PCA), partial least squares discriminant analysis (PLS–DA), and orthogonal partial least squares discriminant analysis (OPLS–DA) to explore treatment-associated metabolic changes within each treatment group (pre- vs. post-treatment). These analyses were intended to explore treatment-associated metabolic patterns rather than to establish predictive models or infer treatment effects between groups. Model performance was evaluated using R^2^ (goodness of fit) and Q^2^ (predictive ability) values. The robustness and validity of the OPLS–DA models were further evaluated using permutation testing (*n* = 2000). Because of the relatively small sample size and high dimensionality of metabolomic data, multivariate analysis results were interpreted cautiously to minimize the risk of model overfitting. Differential metabolites were identified based on variable importance in projection (VIP) scores ≥ 1.0, fold change (FC) values ≥ 1.2 or ≤0.83, and a false discovery rate (FDR)-adjusted *p* < 0.05 using the Benjamini–Hochberg correction method. Receiver operating characteristic (ROC) curve analysis and area under the curve (AUC) calculations were also performed in MetaboAnalyst 6.0 to evaluate the discriminatory performance of candidate metabolites associated with treatment-related metabolic alterations and adjunctive H_2_ gas inhalation. The reported ROC AUC values were derived from the same dataset used for metabolite selection and therefore represent in-sample exploratory estimates without external validation or cross-validation. An AUC ≥ 0.7 was considered indicative of acceptable exploratory discriminatory performance. Pathway enrichment and metabolite set enrichment analyses were performed using the Kyoto Encyclopedia of Genes and Genomes (KEGG) database [16,17,18,19]. To further evaluate between-group differences in treatment-associated changes in the principal metabolite identified in this study (uric acid), within-subject changes (Δ = post-treatment − pre-treatment) were calculated for each participant. This targeted analysis was performed to complement the untargeted metabolomic findings and to directly compare treatment-associated changes between groups. Between-group comparisons of Δ values were performed using the Mann–Whitney U test.

## 3. Results

### 3.1. Characteristics of the Participants

As shown in Figure 1, a total of 31 patients were assessed for eligibility, of whom 20 were randomized into two groups: CCRT alone (Group A, *n* = 10) and CCRT combined with H_2_ gas inhalation (Group B, *n* = 10). One patient in Group B discontinued all treatments at the 14th fraction of radiotherapy after withdrawing consent to pursue alternative herbal treatment. Treatment compliance, acute CCRT-related toxicities, and serum metabolomic analyses were therefore evaluated in 10 and 9 patients in Groups A and B, respectively. At baseline, there were no statistically significant differences between Group A (*n* = 10) and Group B (*n* = 10) with respect to age (median 55.5 vs. 59 years, *p* = 0.54), sex distribution (*p* = 0.61), primary tumor site (*p* = 0.67), stage (*p* = 0.47), or chemotherapy regimen (*p* = 0.26), as shown in Table 1. Baseline demographic and clinical characteristics were generally comparable between the two groups, although the small sample size limited the ability to detect subtle differences.

### 3.2. Treatment Compliance and Overall Treatment Time

A total of 19 patients were evaluated (Group A: CCRT alone, *n* = 10; Group B: CCRT + H_2_, *n* = 9). Delayed chemotherapy occurred more frequently in Group A, where 6 of 10 patients experienced treatment delays, compared with 3 of 9 patients in Group B, although this difference did not reach statistical significance. Group B also demonstrated a slightly shorter overall treatment time, with a median of 48.0 days (range 48–49) compared with 51.5 days (range 48–56) in Group A (Table 2). These descriptive findings may indicate better treatment continuity among patients receiving adjunctive H_2_ gas inhalation; however, the small sample size and the absence of statistically significant differences preclude definitive conclusions.

#### 3.2.1. Acute Non-Hematologic Toxicities

Mild to moderate radiation dermatitis was observed in both groups. In Group A, 6 patients developed Grade 1 and 4 developed Grade 2 dermatitis. In Group B, 7 patients experienced Grade 1 and 2 experienced Grade 2 dermatitis. No Grade 3 dermatitis was observed in either group. Pharyngitis was predominantly low-grade in both groups. In Group A, 7 patients developed Grade 1 and 3 developed Grade 2 pharyngitis, whereas Group B reported 8 Grade 1 and 1 Grade 2 events. No Grade 3 pharyngitis was recorded. The severity of mucositis differed between groups. In Group A, 5 patients developed Grade 1, 4 developed Grade 2, and 1 developed Grade 3 mucositis. In contrast, Group B reported 4 Grade 1 and 5 Grade 2 cases, with no Grade 3 mucositis observed. Overall, acute non-hematologic CCRT-related toxicities were predominantly Grade 1–2 in both groups, with a numerically lower frequency of Grade 2–3 events in the H_2_ gas inhalation group; however, no statistically significant differences were identified between groups (Table 2).

#### 3.2.2. Acute Hematologic Toxicities

Leukopenia patterns differed slightly between groups. In Group A, 3 patients were Grade 0, 1 Grade 1, 4 Grade 2, and 2 Grade 3, whereas Group B showed 4 Grade 0, 3 Grade 1, 2 Grade 2, and no Grade 3 cases, although no statistically significant differences were identified between groups. Neutropenia was predominantly mild in both groups. Group A exhibited 3 Grade 0, 4 Grade 1, 2 Grade 2, and 1 Grade 3 cases, whereas Group B showed 4 Grade 0, 4 Grade 1, 1 Grade 2, and no Grade 3 cases. Thrombocytopenia was uncommon in both groups. Group A had 8 Grade 0 and 2 Grade 1 cases, with no Grade 2 events. Group B had 8 Grade 0 cases and 1 Grade 2 case, with no Grade 1 cases recorded. Overall, hematologic toxicities were predominantly Grade 0–2 in both groups, with few Grade 3 events observed during treatment and no statistically significant differences between groups (Table 2).

At the final follow-up assessment (February 2026), no evidence of disease was documented in 7 patients in Group A and 8 patients in Group B. Locoregional recurrence occurred in 2 patients in Group A and 1 patient in Group B. No distant metastases were observed in either group. One disease-related death occurred in Group A during the follow-up period. However, because of the limited sample size and short follow-up duration, the present study was not designed to evaluate oncological outcomes, and these clinical outcome data are presented descriptively only and should not be interpreted as evidence of differences in recurrence, survival, tumor control, or other oncological outcomes between the study groups.

### 3.3. Serum Metabolomic Profiling

To further characterize treatment-associated metabolic alterations and explore the potential biological responses associated with adjunctive H_2_ gas inhalation, serum metabolomic profiling was performed to characterize CCRT-associated metabolic alterations and to explore the potential metabolic responses associated with adjunctive H_2_ gas inhalation. The serum collection scheme for LAHNC patients in Groups A and B, with samples obtained before and after CCRT in the absence or presence of H_2_ gas inhalation, is shown in Supplementary Figure S1. Comparison of base peak chromatograms revealed differences between pre-and post-treatment samples in both groups, consistent with treatment-associated metabolomic alterations following CCRT. Differences in spectral patterns were also observed in Group B (CCRT + H_2_) compared with Group A (CCRT alone), suggesting possible differences in treatment-associated metabolic patterns between groups; however, these observations were descriptive and should not be interpreted as direct evidence of H_2_-specific metabolic effects.

### 3.4. Multivariate Data Analysis

**Figure 2:**
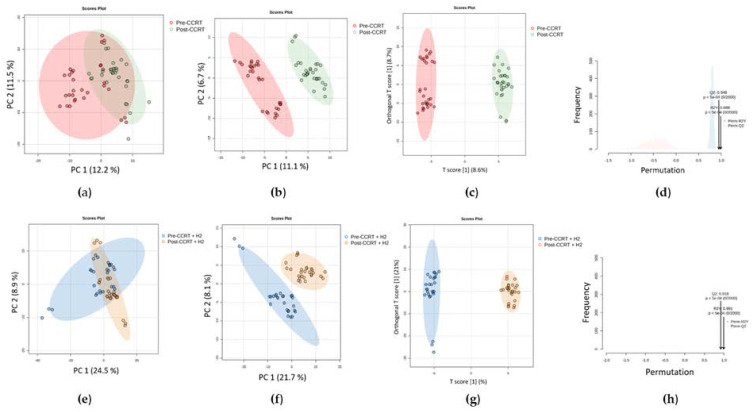
Multivariate statistical analysis. PCA (**a**), PLS–DA (**b**) and OPLS–DA (**c**) score plots comparing serum samples from LAHNC patients collected pre-and post-CCRT (Group A). Permutation testing (*n* = 2000) confirmed the robustness and validity of the OPLS–DA models for Group A is shown in (**d**). PCA (**e**), PLS–DA (**f**) and OPLS–DA (**g**) score plots comparing serum samples from LAHNC patients collected pre-and post-CCRT combined with H_2_ gas inhalation (Group B). Permutation testing (*n* = 2000) confirmed the robustness and validity of the OPLS–DA models for Group B is shown in (**h**).

PCA, PLS–DA and OPLS–DA were performed to evaluate metabolic alterations between serum samples collected before and after CCRT in LAHNC patients in Groups A and B. In Group A (CCRT alone), a total of 385 metabolite features were retained after data preprocessing and quality filtering for subsequent analysis. The OPLS-DA model demonstrated apparent separation between pre- and post-treatment samples (R^2^Y = 0.988, Q^2^ = 0.948), whereas PCA and PLS–DA showed less distinct clustering (Figure 2a–d). Model robustness was assessed using permutation testing (*n* = 2000), which supported the statistical validity of the observed class separation; however, the results should be interpreted cautiously because of the relatively small sample size and the high dimensionality of metabolomic data (*p* < 0.0005). In Group B (CCRT + H_2_ gas inhalation), 389 metabolite features were retained after data preprocessing and quality filtering for subsequent analysis. The OPLS–DA model also demonstrated apparent separation between pre- and post-treatment samples (R^2^Y = 0.991, Q^2^ = 0.918), while PCA and PLS–DA showed less pronounced separation (Figure 2e–h). Permutation testing (*n* = 2000) further supported model stability, although the possibility of partial overfitting cannot be completely excluded because of the limited sample size (*p* < 0.0005). Collectively, the OPLS–DA models suggested separation between pre- and post-treatment samples in both groups, providing supportive exploratory evidence for subsequent differential metabolite and pathway enrichment analyses rather than definitive evidence of treatment effects.

### 3.5. Differential Metabolites and Altered Metabolic Pathways Following CCRT in Patients with LAHNC

**Figure 3:**
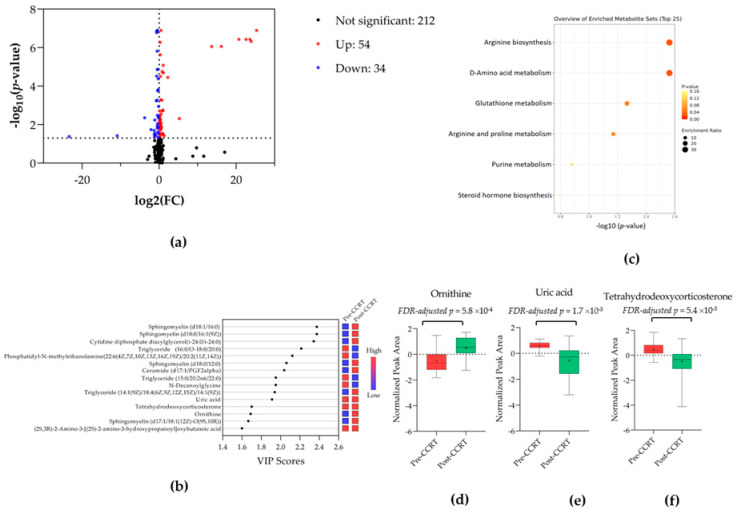
Metabolomic alterations in serum samples collected before and after concurrent chemoradiotherapy (CCRT). (**a**) Volcano plot showing significantly differential metabolites between pre- and post-CCRT samples. Red and blue dots indicate significantly upregulated and downregulated metabolites, respectively, while black dots represent non-significant metabolites. (**b**) Variable importance in projection (VIP) scores of the top 15 discriminative metabolites identified by OPLS–DA analysis. (**c**) KEGG pathway mapping of differential metabolites, illustrating single-hit pathway assignments that provide biological context for the observed metabolite alterations rather than definitive pathway-level perturbations. Representative box plots showing significantly altered metabolites, including ornithine (**d**), uric acid (**e**), and tetrahydrodeoxycorticosterone (**f**), between pre- and post-CCRT groups. Data are presented as normalized peak areas with FDR-adjusted *p* values indicated above each comparison.

Based on the OPLS–DA model, differential metabolite analysis was performed to identify treatment-associated metabolic alterations between pre- and post-CCRT serum samples. Following LC–MS data preprocessing and quality filtering, 385 metabolite features were retained for further statistical analysis. Among these, 54 metabolites were significantly upregulated, 34 were significantly downregulated, and 212 were non-significantly altered, whereas 85 remained unidentified or unannotated (Figure 3a). Applying more stringent selection criteria (VIP > 1.0, FC ≥ 1.2 or ≤0.83, and FDR-adjusted *p* < 0.05), 63 differential metabolite features were identified, comprising 36 features with higher abundance in pre-CCRT samples and 27 features elevated post-treatment (Supplementary Table S1). The top 15 most discriminatory serum metabolite features are presented in Figure 3b, including sphingomyelin, cytidine diphosphate diacylglycerol, phosphatidyl-N-methylethanolamine, ceramide, triglyceride, N-decanoylglycine, uric acid, tetrahydrodeoxycorticosterone, ornithine and (2S,3R)-2-amino-3-[(2S)-amino-3-hydroxypropanoyl]oxybutanoic acid. Several lipid-related metabolites, including sphingomyelins and triglycerides, as well as amino acid-related metabolites, exhibited high VIP scores, suggesting that these metabolic classes contributed substantially to treatment-associated metabolic alterations. Exogenous compounds, unidentified metabolites, and metabolites without well-characterized biological functions were excluded from downstream biological interpretation.

To evaluate the discriminatory potential of these metabolites, ROC analysis was conducted to distinguish between pre-and post-CCRT samples in LAHNC patients. Thirty metabolite features demonstrated exploratory discriminatory performance (AUC ≥ 0.7), with AUC values ranging from 0.70 to 0.92 (95% CI: 0.57–0.98). Among these, lipid-related metabolites predominated, including sphingolipids, glycerophospholipids, and neutral lipids, alongside amino acid and nitrogen metabolism-related metabolites (Table 3). KEGG pathway analysis identified illustrative single-hit pathway assignments that suggest possible involvement of arginine biosynthesis and D-amino acid metabolism (*p* < 0.05). Additional pathways, including glutathione metabolism, arginine and proline metabolism, purine metabolism, and steroid hormone biosynthesis, were represented by illustrative single-hit pathway assignments based on one or a few mapped metabolites (Figure 3c, Table 4).

Notably, ornithine was the only metabolite mapped to multiple KEGG pathways, including arginine biosynthesis, D-amino acid metabolism, glutathione metabolism, and arginine and proline metabolism, suggesting possible involvement of these pathways rather than definitive pathway perturbation. Ornithine levels were significantly increased after treatment compared with pre-treatment (FDR-adjusted *p* = 5.8 × 10^−4^, AUC = 0.78, specificity = 81%, sensitivity = 83%) (Figure 3d, Table 3). In contrast, uric acid levels were significantly decreased after treatment compared with pre-treatment (FDR-adjusted *p* = 1.7 × 10^−5^, AUC = 0.87, specificity = 81%, sensitivity = 87%), which may be associated with purine metabolism and oxidative stress-related responses (Figure 3e, Table 3). Tetrahydrodeoxycorticosterone was also significantly reduced after CCRT compared with pre-treatment (FDR-adjusted *p* = 5.4 × 10^−5^, AUC = 0.79, specificity = 77%, sensitivity = 77%), which may be associated with steroid hormone metabolism (Figure 3f, Table 3). However, these pathway enrichment results should be interpreted cautiously because most enriched pathways were represented by only one or a few mapped metabolites.

### 3.6. Differential Metabolites and Altered Metabolic Pathways Following CCRT Combined with Adjunctive H_2_ Gas Inhalation in Patients with LAHNC

**Figure 4:**
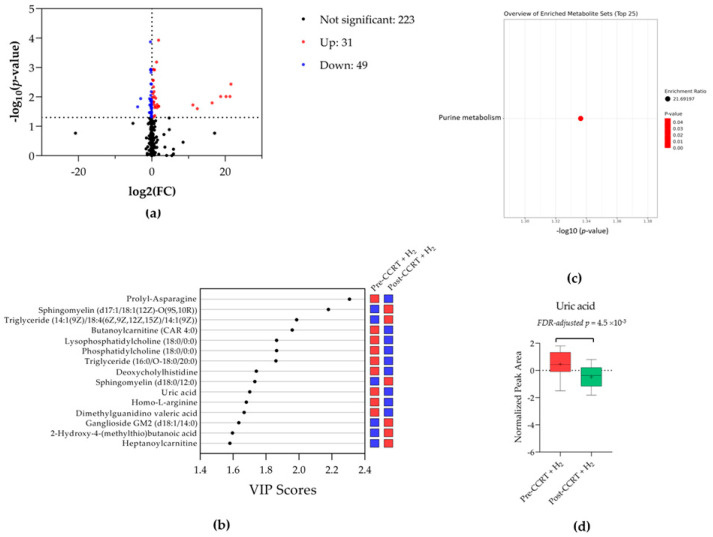
Metabolomic alterations in serum samples collected before and after concurrent chemoradiotherapy (CCRT) combined with H_2_ gas inhalation. (**a**) Volcano plot showing significantly differential metabolites between pre- and post-CCRT combined with H_2_ gas samples. Red and blue dots indicate significantly upregulated and downregulated metabolites, respectively, while black dots represent non-significant metabolites. (**b**) Variable importance in projection (VIP) scores of the top 15 discriminative metabolites identified by OPLS–DA analysis. (**c**) KEGG pathway mapping illustrating an illustrative single-hit pathway assignment based on the differential metabolite uric acid. This mapping is intended to provide biological context rather than definitive evidence of pathway-level perturbation. (**d**) Box plot showing normalized peak area of uric acid in pre- and post-CCRT combined with H_2_ gas samples. Uric acid levels were significantly decreased following treatment (FDR-adjusted *p* = 4.5 × 10^−3^), suggesting alterations in purine metabolism potentially associated with treatment-related oxidative metabolic responses.

Based on the OPLS–DA model described above, differential metabolite analysis was subsequently performed to identify treatment-associated metabolic alterations between pre- and post-treatment serum samples from LAHNC patients receiving CCRT combined with adjunctive H_2_ gas inhalation. Following LC–MS data preprocessing and quality filtering, 389 metabolite features were retained for differential metabolite analysis. Among these, 303 annotated metabolite features were included in the volcano plot analysis, comprising 31 significantly increased, 49 significantly decreased and 223 non-significantly altered metabolites, whereas 86 metabolite features remained unidentified or insufficiently annotated and were therefore excluded from downstream biological interpretation (Figure 4a). Using predefined filtering criteria (VIP > 1.0, FC ≥ 1.2 or ≤0.83, and FDR-adjusted *p* < 0.05), 43 differential metabolite features were identified. Among these, 30 metabolite features exhibited higher relative abundance prior to treatment, while 13 metabolites showed increased abundance after CCRT combined with H_2_ gas inhalation (Supplementary Table S2). The top 15 candidate metabolites ranked by VIP score are presented in Figure 4b. After excluding exogenous compounds and unidentified metabolites to minimize potential non-biological influences, the remaining discriminatory metabolites primarily included lipid-related and amino acid-related metabolites, including sphingomyelins, triglycerides, phosphatidylcholines, lysophosphatidylcholines, prolyl-asparagine, butanoylcarnitine, homo-L-arginine, and uric acid. Several lipid-related metabolites, including sphingomyelins, triglycerides, phosphatidylcholines, and lysophosphatidylcholines, demonstrated high VIP scores, suggesting possible alterations in lipid-related metabolic responses following CCRT.

ROC analysis was conducted to evaluate the discriminatory performance of these metabolites between pre- and post-CCRT combined with H_2_ gas inhalation samples in patients with LAHNC (Table 5). A total of 18 metabolite features demonstrated exploratory discriminatory performance, with in-sample AUC values ranging from 0.70 to 0.88 (95% CI: 0.58–0.95). The identified metabolites were primarily associated with lipid metabolism and amino acid metabolism, with several metabolites previously implicated in oxidative stress-related responses. Furthermore, KEGG pathway mapping identified an illustrative single-hit pathway assignment suggesting possible involvement of purine metabolism (Figure 4c). This pathway assignment was based on the mapping of uric acid (*p* = 0.04), which was significantly decreased after treatment compared with pre-treatment (Figure 4d, Table 5). Uric acid also demonstrated moderate discriminatory performance (FDR-adjusted *p* = 4.5 × 10^−3^, AUC = 0.79, specificity = 74%, sensitivity = 70%). As uric acid is a major end product of purine catabolism, these findings may reflect metabolic changes associated with purine metabolism and oxidative stress-related responses following CCRT combined with H_2_ gas inhalation. Because uric acid consistently emerged as one of the most prominent treatment-associated metabolites across both treatment groups, direct between-group comparison of treatment-associated changes in serum uric acid was subsequently performed to further evaluate the effect of adjunctive H_2_ gas inhalation (Section 3.7). However, these pathway mappings should be interpreted cautiously because the pathway assignment was based on a single mapped metabolite and is intended to provide biological context rather than definitive evidence of pathway-level perturbation. Furthermore, comprehensive direct between-group metabolomic comparisons were beyond the scope of the present analysis.

### 3.7. Direct Comparison of Within-Subject Changes in Serum Uric Acid Between Treatment Groups

Because uric acid was consistently identified as a key differential metabolite in both treatment groups and was the principal metabolite contributing to the exploratory enrichment of purine metabolism, a direct between-group comparison of within-subject changes in serum uric acid (Δ = post-treatment − pre-treatment) was performed to further explore whether treatment-associated changes differed between groups. Although the H_2_-treated group exhibited a numerically smaller reduction in serum uric acid levels than the CCRT-alone group, the between-group difference was not statistically significant (*p* = 0.11; Table 6). These findings suggest that adjunctive H_2_ gas inhalation may be associated with a numerically smaller reduction in serum uric acid levels; however, they do not provide evidence of an H_2_-specific effect on purine metabolism and should therefore be interpreted as exploratory. Although this observation coincided with a numerically lower frequency of moderate treatment-related toxicities in the H_2_-treated group, no causal relationship can be inferred, and confirmation in larger, adequately powered studies is required.

## 4. Discussion

In our previous pilot study, H_2_ gas inhalation administered during CCRT was feasible and well tolerated in patients with LAHNC, with no toxicities attributable to H_2_ and an acceptable safety profile [9]. In the present randomized pilot study, 19 patients with LAHNC were evaluated after receiving either CCRT alone or CCRT combined with adjunctive H_2_ gas inhalation (Table 1, Figure 1). Moderate treatment-related toxicities, including dermatitis, pharyngitis, mucositis, leukopenia, and neutropenia, were observed in both groups, with numerically fewer events observed in the H_2_ group. At the February 2026 follow-up, no disease-related deaths had occurred among patients in the H_2_ group during the available follow-up. However, because of the limited sample size and short follow-up duration, these clinical outcome data are presented for descriptive purposes only and should not be interpreted as evidence of differences in oncological outcomes between the study groups (Table 2). Although the study was not designed to evaluate treatment efficacy or survival outcomes, these preliminary findings support the feasibility and short-term tolerability of adjunctive H_2_ gas inhalation during CCRT. Larger studies with longer follow-up are required to determine its clinical efficacy and long-term oncological outcomes.

Previous studies have similarly reported that H_2_ gas inhalation is safe and may attenuate radiation-induced tissue injury without compromising antitumor efficacy. For example, H_2_ gas inhalation reduced the severity of acute radiation enteritis in cervical cancer patients undergoing CCRT [19]. Mechanistically, H_2_ is recognized as a selective antioxidant capable of neutralizing highly reactive ^•^OH and peroxynitrite (ONOO^−^), while preserving physiologically important ROS involved in normal cellular signaling [20]. In addition to antioxidant effects, H_2_ has been reported to exert anti-inflammatory and cytoprotective actions in normal tissues exposed to oxidative stress [4,5,20]. The timing and duration of H_2_ exposure may influence its biological effects. In the present study, H_2_ inhalation was administered for 1 h daily, approximately 1–2 h prior to irradiation due to clinical workflow constraints. Given the rapid diffusion and systemic distribution of inhaled H_2_ gas [21,22], optimization of inhalation timing relative to CCRT delivery may further influence its potential oxidative stress–modulating effects and warrants investigation in future studies.

Despite the observed differences in toxicity profiles, the underlying biological mechanisms remain incompletely understood. Accordingly, we used exploratory untargeted UHPLC-IM-QTOF-HRMS serum metabolomics to characterize treatment-associated metabolic alterations in patients with LAHNC undergoing CCRT with or without adjunctive H_2_ gas inhalation. The observed alterations in amino acid, lipid, and purine metabolism following CCRT may reflect systemic metabolic responses associated with treatment-related stress and toxicity [23,24]. Among the altered metabolites, ornithine was illustratively mapped to several KEGG pathways, including arginine biosynthesis, D-amino acid metabolism, glutathione metabolism, and arginine and proline metabolism (Figure 3c). Ornithine (Figure 3d) is a key intermediate in arginine metabolism and serves as a precursor for polyamine biosynthesis [25,26]. Increased ornithine levels after CCRT compared with pre-CCRT may be associated with activation of arginine catabolism and polyamine-related pathways in response to treatment-induced cellular stress. Altered ornithine homeostasis has also been associated with inflammation and cellular stress responses [25,26,27,28]. Alterations in sphingomyelins and triglycerides may further indicate changes in membrane lipid turnover and cellular injury responses, as sphingolipid and triglyceride pathways are closely involved in membrane remodeling, inflammatory and stress-response signaling, and cell survival signaling in cancer cells [29,30]. Previous studies have demonstrated that radiation exposure can alter sphingolipid pathways, particularly ceramide-related signaling, thereby contributing to apoptosis and inflammatory responses [31]. In addition, reduced uric acid levels observed after CCRT compared with pre-CCRT may reflect metabolic changes related to purine metabolism (Figure 3e), as uric acid is a major end product of purine catabolism and has been reported to exhibit antioxidant properties under certain physiological conditions [32]. Accordingly, the reduction in uric acid may represent a metabolic response to CCRT-associated oxidative stress rather than a specific biomarker of H_2_ inhalation [32,33,34]. However, because serum uric acid is strongly influenced by renal function, hydration status, diet, and tumor burden, and cisplatin may impair renal function, these factors should also be considered potential confounders when interpreting the observed changes in uric acid [32,35]. A decreasing trend in tetrahydrodeoxycorticosterone was also observed following CCRT (Figure 3f), which may be associated with changes in steroid-related metabolism; however, this observation was supported by a limited number of mapped metabolites, and its biological significance remains unclear and should be interpreted cautiously [30]. Collectively, these findings suggest that metabolic remodeling occurs during CCRT and may accompany systemic inflammatory and cellular stress responses in patients with LAHNC. Accordingly, the biological interpretations presented here should be regarded as hypothesis-generating rather than definitive.

Given the proposed antioxidant and cytoprotective properties of H_2_, metabolomic alterations observed in the H_2_ gas inhalation group were further compared with those in the CCRT-alone group [2,3,4,5]. Interestingly, KEGG pathway mapping identified an illustrative single-hit pathway assignment suggesting possible involvement of purine metabolism following treatment in the H_2_ gas inhalation group (Figure 4c). Although this pathway assignment was based on a single mapped metabolite, purine metabolism is biologically linked to antioxidant defense and cellular stress regulation. Therefore, the more limited metabolic alterations observed in this group may be consistent with partial modulation of CCRT-associated biological responses [20,32,35,36,37,38]. The observed reduction in uric acid levels after CCRT compared with pre-CCRT (Figure 4d) may reflect metabolic changes related to purine metabolism that remained detectable despite adjunctive H_2_ gas inhalation [20,22,32]. However, direct comparisons between treatment groups should be interpreted cautiously because the present metabolomic analyses were performed within each group separately.

Although reduced uric acid levels were observed after CCRT in both treatment groups, the reduction appeared less pronounced in the H_2_-treated group (Figure 3e and Figure 4d). This less pronounced reduction in uric acid may be consistent with partial modulation of treatment-associated oxidative stress and related metabolic responses. To further explore this observation, we compared the within-subject change (Δ = post − pre) in uric acid between the two treatment groups. Although the H_2_-treated group exhibited a numerically smaller reduction in uric acid than the CCRT-alone group, the between-group difference did not reach statistical significance (*p* = 0.11, Table 6). Therefore, these findings should be regarded as exploratory rather than definitive evidence of an H_2_-specific metabolic effect. Notably, the numerically smaller reduction in uric acid coincided with a numerically lower frequency of moderate treatment-related toxicities; however, no causal relationship can be inferred, and this observation requires confirmation in larger, adequately powered prospective studies.

Overall, the present metabolomic findings suggest that CCRT is associated with systemic metabolic remodeling. These pathway-level interpretations should be considered illustrative because several pathway assignments were based on one or a few mapped metabolites and were intended to provide biological context rather than definitive evidence of pathway-level perturbation. However, any additional influence of adjunctive H_2_ gas inhalation remains exploratory because comprehensive between-group comparisons of metabolomic profiles were not performed, and the direct comparison of uric acid changes between treatment groups did not reach statistical significance [22,34,38,39]. The present findings should also be interpreted in the context of the ongoing debate regarding the biological mechanisms of H_2_. Although H_2_ has been proposed to reduce oxidative damage through selective interactions with highly reactive oxidants, its protective effects are unlikely to result solely from direct radical scavenging [3,4,5,6]. Emerging preclinical and limited clinical evidence suggests that modulation of redox signaling, inflammatory responses, mitochondrial function, and cellular stress adaptation may also contribute to its biological activity [4,5,7,8,9]. Furthermore, H_2_ should currently be regarded as a potential adjunctive supportive strategy rather than a universal radioprotective intervention. Future adequately powered prospective studies integrating comprehensive metabolomics with clinical outcomes are warranted to clarify the biological mechanisms of H_2_ and determine whether metabolic alterations are associated with clinically meaningful reductions in treatment-related toxicities without compromising tumor control.

Several limitations should be acknowledged. The relatively small sample size limits statistical power and generalizability, and validation in larger, independent cohorts is required. Another limitation is the heterogeneity in tumor site, stage, chemotherapy regimen, and patient characteristics, which may have influenced metabolic responses and treatment-related toxicities. Although baseline chemotherapy regimens were not statistically different between the two groups, the proportion of patients receiving carboplatin was slightly higher in the H_2_ group. Because cisplatin and carboplatin have distinct toxicity profiles and may differentially influence systemic metabolic responses, this imbalance represents a potential confounding factor. Therefore, the observed clinical and metabolomic findings should be interpreted with caution, and residual confounding cannot be excluded despite generally comparable baseline characteristics. Furthermore, comprehensive metabolomic analyses were performed separately within each treatment group (pre- vs. post-treatment), whereas direct between-group comparison was limited to the principal metabolite (uric acid). Consequently, potential effects specifically attributable to adjunctive H_2_ gas inhalation remain uncertain and require confirmation through direct between-group metabolomic comparisons in larger independent cohorts. In addition, pathway-level interpretations should be considered exploratory because several pathway assignments were based on only one or a few mapped metabolites; therefore, these findings should be regarded as hypothesis-generating. Notably, the pathway assignment for purine metabolism was primarily driven by uric acid, further limiting mechanistic inference. Exogenous compounds and unidentified metabolites were excluded from biological interpretation to reduce potential non-biological influences, although this approach may also have limited pathway coverage. Furthermore, because metabolomic profiling was performed using ESI+ mode only, metabolites that preferentially ionize in negative mode, including several organic acids, may have been underrepresented. Because uric acid was the principal metabolite identified in the present study and is preferentially detected in negative-ion mode, the robustness of its quantification should also be interpreted with appropriate caution. Accordingly, the present metabolomic dataset may not fully capture all treatment-related metabolic alterations, and future studies incorporating both positive and negative ionization modes are warranted to achieve more comprehensive metabolomic coverage. In addition, because authentic reference standards were not analyzed and CCS information was not used for metabolite annotation, the identities of the reported discriminatory metabolites should be regarded as putative annotations and interpreted with appropriate caution. Although several metabolites demonstrated relatively high in-sample discriminatory performance, external validation was not performed. Furthermore, because the ROC analyses were performed using the same dataset employed for metabolite discovery, the reported diagnostic performance is likely to be optimistic until validated in independent cohorts. Similarly, although permutation testing supported the robustness of the OPLS–DA models, the relatively small sample size and lack of external validation increase the risk of model overfitting, and the multivariate findings should therefore be interpreted cautiously. Finally, individual factors such as baseline body composition, nutritional status, hydration, renal function, and intrinsic radiosensitivity, together with the relatively short duration and fixed once-daily timing of H_2_ gas inhalation, may have influenced systemic metabolomic profiles, treatment tolerance, and the extent of detectable metabolic modulation during CCRT. Future adequately powered multicenter prospective studies incorporating longitudinal sampling, comprehensive between-group metabolomic analyses, external validation, and mechanistic investigations are warranted to clarify the biological and clinical significance of adjunctive H_2_ gas inhalation during CCRT.

## 5. Conclusions

H_2_ gas inhalation during CCRT was generally well tolerated in patients with LAHNC and may be associated with a lower frequency of moderate treatment-related toxicities, although the present pilot study was not designed to evaluate oncological outcomes. Untargeted serum metabolomic profiling revealed exploratory alterations in metabolites linked to purine metabolism and lipid-related pathways following adjunctive H_2_ gas inhalation, with uric acid emerging as a key treatment-associated metabolite that may be associated with oxidative stress-related metabolic responses during CCRT. Although the H_2_-treated group exhibited a numerically smaller reduction in uric acid levels than the CCRT-alone group, direct between-group comparison of within-subject changes did not demonstrate a statistically significant difference. Accordingly, these findings are consistent with the hypothesis that adjunctive H_2_ gas inhalation may be associated with differences in oxidative stress-related metabolic responses during CCRT. However, because the reported pathway assignments were based on only a limited number of mapped metabolites and the discriminatory performance of individual metabolites was not externally validated, these findings should be considered hypothesis-generating rather than definitive. In addition, the relatively small sample size and heterogeneous patient population further limit the generalizability of the present findings. Further validation in larger, independent cohorts using comprehensive between-group metabolomic analyses is warranted.

## Figures and Tables

**Table 1:** Patient and treatment characteristics.

Variables	Group A (CCRT) (*n* = 10)	Group B (CCRT + H2) (*n* = 10)	*p* Value
**Age**			0.54
Median	55.5	59	
Range	46–64	52–65	
**Sex**			0.61
Male	3	3	
Female	7	7	
**Primary tumor**			0.67
Nasopharynx	2	2	
Oropharynx	1	3	
Hypopharynx	3	1	
Larynx	0	1	
Oral cavity	3	2	
Nasal cavity	1	1	
**Stage**			0.47
III	1	3	
IVA	5	4	
IVB	4	3	
**Chemotherapy regimen**			0.26
Cisplatin	9	7	
Carboplatin	1	3	

**Table 2:** Compliance of treatment and acute toxicities.

Variables	Group A (CCRT) (*n* = 10)	Group B (CCRT + H2) (*n* = 9)	*p* Value
**Delayed chemotherapy**			0.25
No	4	6	
YES	6	3	
**Overall treatment time**; Median (Range) (days)	51.5 (48–56)	48.0 (48–49)	0.15
**Dermatitis**			0.41
Grade 1	6	7	
Grade 2	4	2	
**Pharyngitis**			0.31
Grade 1	7	8	
Grade 2	3	1	
**Mucositis**			0.56
Grade 1	5	4	
Grade 2	4	5	
Grade 3	1	0	
**Leucopenia**			0.29
Grade 0	3	4	
Grade 1	1	3	
Grade 2	4	2	
Grade 3	2	0	
**Neutropenia**			0.79
Grade 0	3	4	
Grade 1	4	4	
Grade 2	2	1	
Grade 3	1	0	
**Thrombocytopenia**			0.23
Grade 0	8	8	
Grade 1	2	0	
Grade 2	0	1	
**Status at the last follow-up**; (February 2026)			0.51
No evidence of disease	7	8	
Locoregional recurrence	2	1	
Distant metastasis	0	0	
Dead of disease	1	0	

**Table 3:** Differential serum metabolites identified between pre- and post-CCRT samples in patients with locally advanced head and neck cancer.

Metabolites	VIP	FC	FDR-Adjusted *p*	Direction	AUC	C1	C2	S1	S2
1. Sphingomyelin (d18:0/16:1(9Z))	2.38	0.72	1.3 × 10^−7^	Down	0.92	0.82	0.97	0.87	0.87
2. Sphingomyelin (d18:1/16:0)	2.38	0.72	1.3 × 10^−7^	Down	0.92	0.84	0.98	0.87	0.87
3. Cytidine diphosphate diacylglycerol(i-24:0/i-24:0)	2.37	0.65	1.4 × 10^−7^	Down	0.87	0.76	0.95	0.87	0.77
4. Phosphatidyl-N-methylethanolamine(22:6(4Z,7Z,10Z,13Z,16Z,19Z)/20:2(11Z,14Z))	2.13	1.22	2.3 × 10^−6^	Up	0.85	0.74	0.94	0.81	0.87
5. Sphingomyelin (d18:0/12:0)	2.08	0.73	1.4 × 10^−7^	Down	0.84	0.71	0.92	0.74	0.80
6. Ceramide (d17:1/PGF2alpha)	2.06	0.74	1.4 × 10^−7^	Down	0.85	0.74	0.94	0.77	0.73
7. N-Decanoylglycine	1.96	2.13	2.1 × 10^−5^	Up	0.85	0.74	0.93	0.90	0.77
8. Uric acid	1.92	1.70	1.7 × 10^−5^	Up	0.87	0.77	0.96	0.81	0.87
9. Tetrahydrodeoxycorticosterone	1.71	1.66	5.4 × 10^−5^	Up	0.79	0.65	0.89	0.77	0.77
10. Ornithine	1.70	0.61	5.8 × 10^−4^	Down	0.78	0.66	0.89	0.81	0.83
11. Sphingomyelin (d17:1/18:1(12Z)-O(9S,10R))	1.66	0.69	5.4 × 10^−4^	Down	0.79	0.67	0.91	0.81	0.67
12. (2S,3R)-2-Amino-3-[(2S)-2-amino-3-hydroxypropanoyl]oxybutanoic acid	1.62	1.30	5.1 × 10^−4^	Up	0.78	0.64	0.88	0.84	0.60
13. 5-amino-1-formylimidazole-4-carbonitrile	1.56	1.92	1.8 × 10^−3^	Up	0.75	0.64	0.87	0.61	0.80
14. N(5)-Acetylornithine	1.55	1.95	1.9 × 10^−3^	Up	0.77	0.65	0.87	0.61	0.80
15. Deoxycholylhistidine	1.50	1.43	2.8 × 10^−3^	Up	0.73	0.60	0.83	0.84	0.60
16. Ganglioside GM2 (d18:1/14:0)	1.46	0.46	6.0 × 10^−3^	Down	0.80	0.69	0.89	0.84	0.63
17. Lysophosphatidylcholine (18:0/0:0)	1.43	1.20	7.3 × 10^−3^	Up	0.76	0.63	0.87	0.81	0.63
18. Phosphatidylcholine (18:0/0:0)	1.43	1.20	7.3 × 10^−3^	Up	0.76	0.63	0.88	0.81	0.63
19. 3-Palmitoyl-sn-glycerol	1.40	1.26	8.2 × 10^−3^	Up	0.80	0.68	0.90	0.87	0.73
20. L-Cystine	1.36	0.77	9.0 × 10^−3^	Down	0.71	0.57	0.83	0.52	0.93
21. Phosphatidic Acid (20:4(6E,8Z,11Z,14Z)-OH(5S)/17:0)	1.34	1.57	0.013	Up	0.70	0.57	0.83	0.61	0.70
22. 1-Methylhistidine	1.34	1.36	0.012	Up	0.73	0.59	0.84	0.77	0.60
23. Creatinine	1.34	1.27	9.8 × 10^−3^	Up	0.77	0.65	0.87	0.81	0.63
24. L-2-Amino-3-(1-pyrazolyl)propanoic acid	1.23	1.22	0.019	Up	0.72	0.58	0.87	0.90	0.70
25. 8-Oxo-7,8-dihydrodeoxyguanine	1.17	1.95	0.028	Up	0.72	0.57	0.83	0.52	0.90
26. N-(2-hydroxyhexadecanoyl)-4-hydroxy-15-methylhexadecasphinganine-1-phosphocholine	1.17	0.80	2.4 × 10^−3^	Down	0.71	0.57	0.84	0.77	0.63
27. (5Z,8Z,11Z,14Z,17Z)-Icosa-5,8,11,14,17-pentaenoylcarnitine	1.12	0.43	0.045	Down	0.81	0.69	0.90	0.74	0.87
28. Arabinosylhypoxanthine	1.11	2.48	0.038	Up	0.73	0.58	0.85	0.68	0.70

**Notes**: VIP, variable importance in projection; FC, fold change [FC was calculated as the ratio of pre-treatment to post-treatment metabolite abundance (Pre/Post)]; FDR-adjusted *p* value, false discovery rate-adjusted probability value; Direction refers to metabolite abundance relative to the pre-treatment samples (Up = higher in Pre-CCRT; Down = higher in Post-CCRT); AUC, area under the ROC curve; C1 and C2, lower and upper limits of the 95% confidence interval, respectively; S1, specificity; S2, sensitivity.

**Table 4:** KEGG pathway mapping of differential serum metabolites before and after concurrent chemoradiotherapy, illustrating single-hit pathway assignments.

Pathways	Total	Hits	Hits Compound	*p* Value
1. Arginine biosynthesis	14	1	Ornithine	0.027
2. D-Amino acid metabolism	14	1	Ornithine	0.027
3. Glutathione metabolism	28	1	Ornithine	0.054
4. Arginine and proline metabolism	35	1	Ornithine	0.067
5. Purine metabolism	70	1	Uric acid	0.132
6. Steroid hormone biosynthesis	86	1	Tetrahydrodeoxycorticosterone	0.161

**Table 5:** Differential serum metabolites identified between pre- and post-CCRT combined with H_2_ gas inhalation samples in patients with locally advanced head and neck cancer.

Metabolites	VIP	FC	FDR-Adjusted *p*	Direction	AUC	C1	C2	S1	S2
1. Prolyl-Asparagine	2.31	3.56	1.2 × 10^−4^	Up	0.88	0.78	0.95	0.78	0.85
2. Sphingomyelin (d17:1/18:1(12Z)-O(9S,10R))	2.18	0.76	1.3 × 10^−5^	Down	0.84	0.72	0.94	0.85	0.78
3. Triglyceride (14:1(9Z)/18:4(6Z,9Z,12Z,15Z)/14:1(9Z))	1.99	0.82	1.1 × 10^−3^	Down	0.83	0.69	0.94	0.85	0.89
4. Butanoylcarnitine (CAR 4:0)	1.96	1.61	1.2 × 10^−3^	Up	0.82	0.69	0.93	0.78	0.89
5. Lysophosphatidylcholine (18:0/0:0)	1.86	1.29	2.7 × 10^−3^	Up	0.83	0.71	0.93	0.70	0.89
6. Phosphatidylcholine (18:0/0:0)	1.86	1.29	2.7 × 10^−3^	Up	0.83	0.70	0.93	0.70	0.89
7. Triglyceride (16:0/O-18:0/20:0) *	1.86	3.1 × 10^6^	3.7 × 10^−3^	Up	0.70	0.61	0.80	1.00	0.41
8. Deoxycholylhistidine	1.74	1.50	6.6 × 10^−3^	Up	0.76	0.61	0.89	0.85	0.67
9. Uric acid	1.70	1.35	4.5 × 10^−3^	Up	0.79	0.66	0.90	0.74	0.70
10. Homo-L-arginine	1.68	1.57	9.7 × 10^−3^	Up	0.78	0.66	0.89	0.85	0.56
11. Dimethylguanidino valeric acid	1.66	2.06	0.011	Up	0.75	0.61	0.87	0.67	0.70
12. Heptanoylcarnitine	1.58	0.68	0.018	Down	0.72	0.58	0.85	0.74	0.63
13. N-(2-Hydroxypropyl)valine	1.57	1.37	0.015	Up	0.77	0.62	0.89	0.85	0.63
14. Acetyl-L-carnitine (CAR 2:0)	1.48	3.57	0.021	Up	0.77	0.63	0.88	0.67	0.78
15. 1-Methylhistidine	1.47	1.42	0.015	Up	0.76	0.62	0.88	0.78	0.67
16. L-Histidinol	1.43	1.40	0.018	Up	0.76	0.61	0.89	0.78	0.67
17. 8-Oxo-7,8-dihydrodeoxyguanine	1.42	2.66	0.023	Up	0.78	0.65	0.90	0.70	0.67
18. N(5)-Acetylornithine	1.41	1.76	0.023	Up	0.77	0.62	0.89	0.70	0.96

**Notes**: VIP, variable importance in projection; FC, fold change [FC was calculated as the ratio of pre-treatment to post-treatment metabolite abundance (Pre/Post)]; FDR-adjusted *p* value, false discovery rate-adjusted probability value; Direction refers to metabolite abundance relative to the pre-treatment samples (Up = higher in Pre-CCRT; Down = higher in Post-CCRT); AUC, area under the ROC curve; C1 and C2, lower and upper limits of the 95% confidence interval, respectively; S1, specificity; S2, sensitivity; * This metabolite exhibited an extremely high fold change because of very low abundance in one comparison group and should therefore be interpreted with caution.

**Table 6:** Direct comparison of within-subject changes (Δ = post-treatment − pre-treatment) in serum uric acid levels between the CCRT-alone group and the CCRT plus adjunctive H_2_ gas inhalation group.

Parameter	Group A (CCRT)	Group B (CCRT + H_2_)	*p* Value
Δ Uric acid			0.11
Mean	−39,917.15	−24,039.56	
SD	48,873.64	13,924.30	

## Data Availability

The data presented in this study are available upon request from the corresponding author.

## Supplementary Materials

The following supporting information can be downloaded at https://www.mdpi.com/article/10.3390/cancers18142191/s1. Figure S1: Represent chromatogram of serum obtained from LAHNC patients. Group A: LAHNC patients pre-CCRT (a) and post-CCRT (b). Group B: LAHNC patients pre-CCRT (c) and post-CCRT (d) combined with H_2_ gas inhalation. Table S1: Differential metabolites distinguishing pre- and post-concurrent chemoradiotherapy (CCRT) samples in patients with locally advanced head and neck cancer (LAHNC) (VIP ≥ 1, FC ≥ 1.2 or ≤0.83, and FDR-adjusted *p* < 0.05). Table S2: Differential metabolites distinguishing pre- and post-concurrent chemoradiotherapy combined with hydrogen gas inhalation (CCRT + H_2_) samples in patients with locally advanced head and neck cancer (LAHNC) (VIP ≥ 1, FC ≥ 1.2 or ≤0.83, and FDR-adjusted *p* < 0.05).

## Abbreviations

AUC	Area Under the Curve
CCRT	Concurrent Chemoradiotherapy
CCS	Collision Cross Section
CI	Confidence Interval
ECOG	Eastern Cooperative Oncology Group
ESI	Electrospray Ionization
FC	Fold Change
HMDB	Human Metabolome Database
HRMS	High-Resolution Mass Spectrometry
IM	Ion Mobility
IMRT	Intensity Modulated Radiotherapy
KEGG	Kyoto Encyclopedia of Genes and Genomes
LAHNC	Locally Advanced Head and Neck Cancer
LC	Liquid Chromatography
METLIN	Metabolite and Tandem Mass Spectrometry
MS	Mass Spectrometry
MSI	Metabolomics Standards Initiative
OPLS-DA	Orthogonal Partial Least Squares-Discriminate Analysis
PCA	Principal Component Analysis
PLS-DA	Partial Least Squares-Discriminate Analysis
QC	Quality Control
QTOF	Quadrupole Time-of-Flight
ROC	Receiver Operating Characteristic
ROS	Reactive Oxygen Species
RT	Radiotherapy
SD	Standard Deviation
SiMD	Siriraj Metabolomics Data Warehouse
TOF	Time-of-Flight
VIP	Variable Importance in the Projection
UHPLC	Ultra-High-Performance Liquid Chromatography
