# Supplementary material for: Metabolomic Alterations Associated with Adjunctive Hydrogen Gas Inhalation During Concurrent Chemoradiotherapy in Locally Advanced Head and Neck Cancer: A Pilot Study
Source: Cancers (Basel). 2026 Jul 8;18(14):2191. doi: 10.3390/cancers18142191 (PMC13407243; doi:10.3390/cancers18142191)
Supplement: Supplementary file 1 [file cancers-18-02191-s001.zip › cancers-4373551-supplementary.pdf]

Supplementary Materials for

Metabolomic Alterations Associated with Hydrogen Gas Inhalation during Concurrent Chemoradiotherapy in Patients with Locally Advanced Head and Neck Cancer: A Pilot Study

Imjai Chitapanarux *et al*.

*Corresponding author. Email: narongchai.a@cmu.ac.th

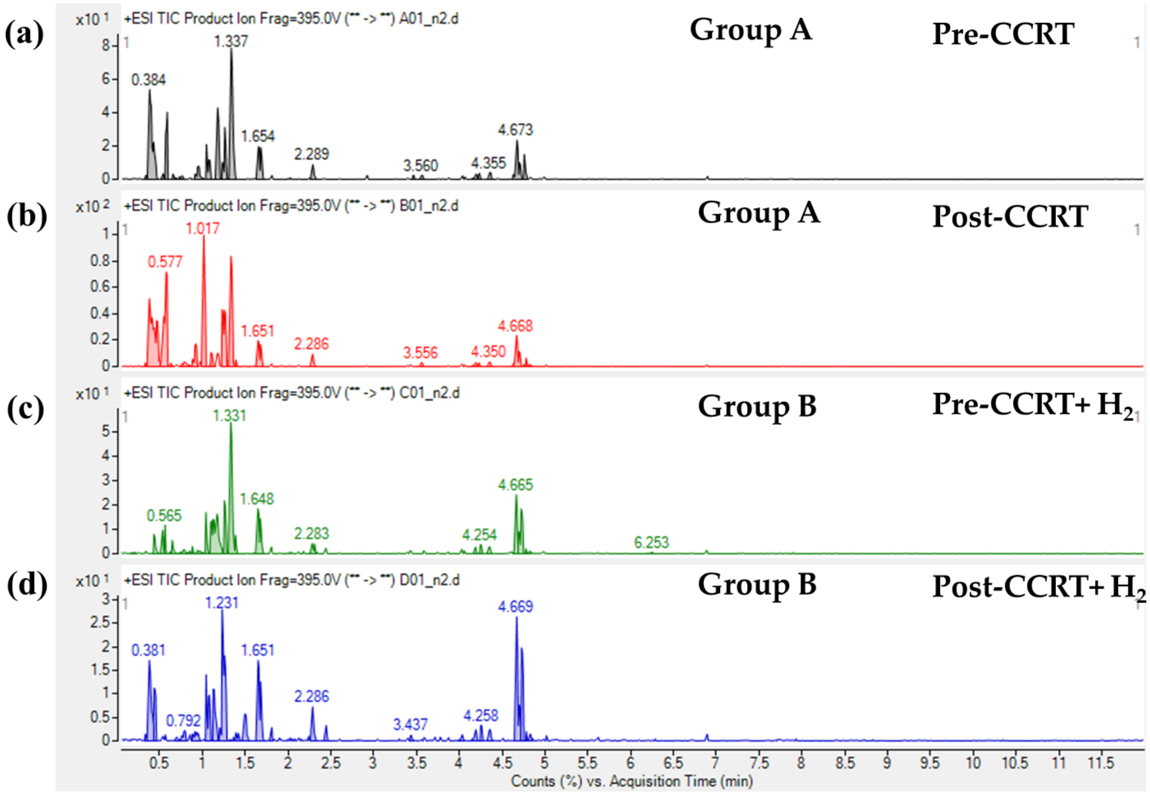

Figure S1. Represent chromatogram of serum obtained from LAHNC patients. Group A: LAHNC patients pre-CCRT (**a**) and post-CCRT (**b**). Group B: LAHNC patients pre-CCRT (**c**) and post-CCRT (**d**) combined with H_2 gas inhalation.

Supplementary Table S1. Differential metabolites distinguishing pre- and post-concurrent chemoradiotherapy (CCRT) samples in patients with locally advanced head and neck cancer (LAHNC) (VIP ≥ 1, FC ≥ 1.2 or ≤ 0.83, and FDR-adjusted $p < 0.05$).

Notes: VIP, variable importance in projection derived from the OPLS–DA model; FC, fold change (pre-CCRT/post-CCRT); FDR–adjusted p value, false discovery rate-adjusted probability value. Direction indicates whether metabolite abundance was higher before or after treatment ((Up = higher in Pre-CCRT; Down = higher in Post-CCRT). Exogenous compounds and unidentified metabolites were excluded from the final analysis.

Metabolites	VIP	FC	*p* value	FDR-adjusted *p*	Direction
1. Iohexol	2.41	4.2×10^{7}	6.2×10^{-10}	1.3×10^{-7}	Up
2.(2R,3R,4R)-2-Amino-4-hydroxy-3-methylpentanoic acid	2.36	1.40	1.3×10^{-9}	1.3×10^{-7}	Up
3. Surotomycin	2.29	6.3×10^{6}	8.5×10^{-9}	3.7×10^{-7}	Up
4. Menaquinol-12	2.29	1.7×10^{6}	8.6×10^{-9}	3.7×10^{-7}	Up
5.5-(Acetylamino)-N,N'-bis(2,3-dihydroxypropyl)-2,4,6-triiodo-1,3-benzenedicarboxamide	2.22	1.2×10^{7}	8.5×10^{-9}	3.7×10^{-7}	Up
6. Alpha-amyrin tetratriacontanoate	2.27	1.6×10^{7}	1.2×10^{-8}	4.8×10^{-7}	Up
7. Triglyceride (16:0/O-18:0/20:0)	2.23	1.3×10^{4}	2.8×10^{-8}	8.6×10^{-7}	Up
8. 1-Phenazinol	2.22	7.3×10^{4}	2.9×10^{-8}	8.6×10^{-7}	Up
9.Phosphatidyl-N-methylethanolamine (22:6(4Z,7Z,10Z,13Z,16Z,19Z)/20:2(11Z,14Z))	2.13	1.22	9.1×10^{-8}	2.3×10^{-6}	Up
10.(16?,20R)-20,24-epoxy-2,16-dihydroxy-25,26,27-trinorcucurbita-1,5,23-triene-3,11,22-trione 2-O-β-D-glucopyranoside	2.02	2.06	3.4×10^{-7}	8.3×10^{-6}	Up
11. Triglyceride (15:0/20:2n6/22:0)	1.97	4.77	2.3×10^{-6}	3.5×10^{-5}	Up
12. N-Decanoylglycine	1.96	2.13	1.2×10^{-7}	2.1×10^{-5}	Up
13. Vanillic acid diethylamide	1.93	1.27	1.9×10^{-6}	3.1×10^{-5}	Up
14. Uric acid	1.92	1.71	9.1×10^{-7}	1.7×10^{-5}	Up
15. Tetrahydrodeoxycorticosterone	1.71	1.66	4.9×10^{-5}	5.4×10^{-4}	Up
16.(2S,3R)-2-Amino-3-[(2S)-2-amino-3-hydroxypropanoyl]oxybutanoic acid	1.62	1.30	4.4×10^{-5}	5.1×10^{-4}	Up
17. 5-amino-1-formylimidazole-4-carbonitrile	1.56	1.92	1.8×10^{-5}	1.8×10^{-3}	Up
18. N(5)-Acetylornithine	1.55	1.95	2.1×10^{-4}	1.9×10^{-3}	Up
19. Deoxycholylhistidine	1.50	1.43	3.1×10^{-4}	2.8×10^{-3}	Up
20. Tromethamine	1.49	37.76	6.6×10^{-4}	4.8×10^{-3}	Up
21. 13-Docosenamide	1.44	1.25	7.7×10^{-4}	5.5×10^{-3}	Up
22. Lysophosphatidylcholine (18:0/0:0)	1.43	1.20	1.1×10^{-3}	7.3×10^{-3}	Up
23. Phosphatidylcholine (18:0/0:0)	1.43	1.20	1.1×10^{-3}	7.3×10^{-3}	Up
24. 6-Aminonicotinamide	1.43	1.25	7.8×10^{-4}	5.5×10^{-3}	Up
25. 3-Palmitoyl-sn-glycerol	1.40	1.26	1.3×10^{-3}	8.2×10^{-3}	Up
26.Phosphatidic Acid (20:4(6E,8Z,11Z,14Z)-	1.34	1.59	2.6×10^{-3}	0.013	Up

OH(5S)/17:0)					
27. 1-Methylhistidine	1.34	1.36	2.3×10^{-3}	0.012	Up
28. Creatinine	1.34	1.27	1.7×10^{-3}	9.8×10^{-3}	Up
29. L-Histidinol	1.33	1.35	2.3×10^{-3}	0.012	Up
30. N6-[2-(4-Aminophenyl)ethyl]adenosine	1.32	1.43	1.4×10^{-3}	0.008	Up
31. Scyphostatin	1.23	1.44	3.5×10^{-3}	0.017	Up
32. L-2-Amino-3-(1-pyrazolyl)propanoic acid	1.23	1.22	4.3×10^{-3}	0.019	Up
33. Ibutilide	1.22	1.24	2.9×10^{-3}	0.015	Up
34. 8-Oxo-7,8-dihydrodeoxyguanine	1.17	1.95	6.9×10^{-3}	0.028	Up
35.6-Acetyl-2,3-dihydro-2-(hydroxymethyl)-4(1H)-pyridinone	1.13	1.93	8.1×10^{-3}	0.033	Up
36. Arabinosylhypoxanthine	1.11	2.48	9.9×10^{-3}	0.038	Up
37. Sphingomyelin (d18:1/16:0	2.38	0.72	9.8×10^{-10}	1.3×10^{-7}	Down
38. Sphingomyelin (d18:0/16:1(9Z))	2.38	0.72	9.8×10^{-10}	1.3×10^{-7}	Down
39.Alpha-D-rhamnosyl-(1?4)-N-acetyl-D-glucosaminyl undecaprenyl diphosphate(2?)	2.37	0.65	2.6×10^{-9}	1.7×10^{-7}	Down
40. Cytidine diphosphate diacylglycerol(i-24:0/i-24:0)	2.37	0.65	1.9×10^{-9}	1.4×10^{-7}	Down
41. Ditrans,polycis-tetradecaprenyl diphosphate(3?)	2.20	0.69	5.6×10^{-8}	1.5×10^{-6}	Down
42. Sphingomyelin (d18:0/12:0)	2.08	0.73	6.7×10^{-7}	1.4×10^{-5}	Down
43. Ceramide (d17:1/PGF2alpha)	2.06	0.74	6.6×10^{-7}	1.4×10^{-5}	Down
44.triglyceride (14:1(9Z)/18:4(6Z,9Z,12Z,15Z)/14:1(9Z))	1.95	0.64	1.8×10^{-6}	3.0×10^{-5}	Down
45. Thrombin Receptor Activator Peptide 6	1.83	0.76	1.4×10^{-5}	1.7×10^{-4}	Down
46. Ornithine	1.70	0.61	5.4×10^{-5}	5.8×10^{-4}	Down
47. Sphingomyelin (d17:1/18:1(12Z)-O(9S,10R))	1.66	0.69	4.8×10^{-5}	5.4×10^{-4}	Down
48. Validamine	1.58	0.74	3.7×10^{-4}	0.003	Down
49. Naphthol AS-BI ?-D-glucuronide	1.56	0.074	5.8×10^{-4}	0.004	Down
50. Ganglioside GM2 (d18:1/14:0)	1.46	0.46	8.4×10^{-4}	0.006	Down
51. L-Cystine	1.36	0.77	1.4×10^{-3}	0.009	Down
52. Tetrachlorophthalic anhydride	1.34	0.71	0.003	0.015	Down
53. Erythrityl Tetranitrate	1.30	0.72	2.1×10^{-3}	0.011	Down
54. Man-?1-6-Ins-1-P-Cer(t20:0/2-OH-26:0)	1.30	0.46	6.6×10^{-3}	0.028	Down
55. Patamostat	1.27	0.41	4.5×10^{-3}	0.020	Down
56. Uzarigenin 3-[xylosyl-(1->2)-rhamnoside]	1.21	0.23	4.0×10^{-3}	0.018	Down
57. (1E,3E)-1-(4-Fluorophenyl)-2-methyl-1-penten-3-one oxime	1.20	0.55	8.9×10^{-3}	0.034	Down
58.8-amino-2,12-dihydroxy-4a,9-dihydro[1,3,2]dioxaphosphinino[4',5':5,6]pyrano[3,2-g]pteridin-10(4H)-one 2-oxide	1.19	0.42	8.5×10^{-3}	0.033	Down
59. 17-epiestriol 17-O-(?-D-glucuronide)(1?)	1.18	5.3×10^{-4}	9.9×10^{-3}	0.038	Down
60.N-(2-hydroxyhexadecanoyl)-4-hydroxy-15-methylhexadecasphinganine-1-phosphocholine	1.17	0.80	5.5×10^{-3}	0.023	Down

61. Bafetinib	1.14	9.4×10^{-8}	0.011	0.042	Down
62.5Z,8Z,11Z,14Z,17Z)-Icosa-5,8,11,14,17-pentaenoylcarnitine	1.12	0.43	0.013	0.045	Down
63. 2-Methyl-1-propenethiol	1.10	0.82	0.012	0.045	Down

Supplementary Table S2. Differential metabolites distinguishing pre- and post-concurrent chemoradiotherapy combined with hydrogen gas inhalation (CCRT+H_2) samples in patients with locally advanced head and neck cancer (LAHNC) (VIP ≥ 1, FC ≥ 1.2 or ≤ 0.83, and FDR-adjusted $p < 0.05$).

Notes: VIP, variable importance in projection derived from the OPLS–DA model; FC, fold change (pre-CCRT/post-CCRT); FDR–adjusted p value, false discovery rate-adjusted probability value. Direction indicates whether metabolite abundance was higher before or after treatment ((Up = higher in Pre-CCRT; Down = higher in Post-CCRT). Exogenous compounds and unidentified metabolites were excluded from the final analysis.

Metabolites	VIP	FC	*p* value	FDR-adjusted *p*	Direction
1. Prolyl-Asparagine	2.31	3.56	3.0×10^{-7}	1.2×10^{-4}	Up
2. Scyphostatin	2.12	2.42	5.0×10^{-6}	6.5×10^{-4}	Up
3.(2S,3R)-2-Amino-3-[(2S)-2-amino-3-hydroxypropanoyl] oxybutanoic acid	2.03	1.25	1.4×10^{-5}	1.1×10^{-3}	Up
4. Butanoylcarnitine (CAR 4:0)	1.96	1.61	2.5×10^{-5}	1.2×10^{-3}	Up
5. 5-aminopentyl ?-L-rhamnoside	1.96	1.61	2.5×10^{-5}	1.2×10^{-3}	Up
6. Lysophosphatidylcholine (18:0/0:0)	1.86	1.29	9.9×10^{-5}	2.7×10^{-3}	Up
7. Phosphatidylcholine (18:0/0:0)	1.86	1.29	9.9×10^{-5}	2.7×10^{-3}	Up
8. Triglyceride (16:0/O-18:0/20:0)	1.86	3.1×10^{6}	1.6×10^{-4}	3.7×10^{-3}	Up
9. Deoxycholylhistidine	1.74	1.50	3.5×10^{-4}	6.6×10^{-3}	Up
10. Iohexol	1.71	2.5×10^{6}	7.0×10^{-4}	9.7×10^{-3}	Up
11.5-(Acetylamino)-N,N'-bis(2,3-dihydroxypropyl)-2,4,6-triiodo-1,3-benzenedicarboxamide	1.71	1.2×10^{6}	7.0×10^{-4}	9.7×10^{-3}	Up
12. Surotomycin	1.71	4.4×10^{5}	7.0×10^{-4}	9.7×10^{-3}	Up
13. Uric acid	1.70	1.35	2.1×10^{-4}	4.5×10^{-3}	Up
14. Homo-L-arginine	1.68	1.57	6.9×10^{-4}	9.7×10-3	Up
15. Dimethylguanidino valeric acid	1.66	2.06	8.6×10^{-4}	0.011	Up
16. L-2-Amino-3-(1-pyrazolyl)propanoic acid	1.66	1.21	4.3×10^{-4}	7.7×10^{-3}	Up
17. Menaquinol-12	1.60	8.8×10^{4}	1.7×10^{-3}	0.016	Up
18. N-(2-Hydroxypropyl)valine	1.57	1.37	0.002	0.015	Up
19. Triglyceride (15:0/20:2n6/22:0)	1.57	2.66	0.002	0.018	Up
20. 1-Phenazinol	1.54	2.4×10^{3}	0.002	0.019	Up
21. Acetyl-L-carnitine (CAR 2:0)	1.48	3.57	0.003	0.021	Up
22.N-[(3s)-2-Oxotetrahydrofuran-3-Yl]butanamide	1.48	3.57	0.003	0.021	Up
23. Alpha-Amyrin tetratriacontanoate	1.48	5.3×10^{3}	0.005	0.025	Up
24. 1-Methylhistidine	1.47	1.42	0.002	0.015	Up
25. L-Histidinol	1.43	1.40	0.002	0.018	Up
26. 6-Hydroxypseudooxynicotine	1.42	1.90	0.002	0.020	Up
27. 8-Oxo-7,8-dihydrodeoxyguanine	1.42	2.66	0.004	0.023	Up

28. N(5)-Acetylornithine	1.41	1.76	0.004	0.023	Up
29. 3,7-bis(dimethylamino)phenothiazin-5-ium	1.30	1.74	0.009	0.043	Up
30. 4-aminobutyl ?-L-fucopyranoside	1.13	1.38	0.012	0.048	Up
31. Sphingomyelin (d17:1/18:1(12Z)-O(9S,10R))	2.18	0.76	6.9×10^{-7}	1.3×10^{-5}	Down
32.triglyceride (14:1(9Z)/18:4(6Z,9Z,12Z,15Z)/14:1(9Z))	1.99	0.82	1.7×10^{-5}	1.1×10^{-3}	Down
33. 5-bromo-4-chloro-3-indolyl acetate	1.97	0.79	3.0×10^{-5}	1.2×10^{-3}	Down
34. Sphingomyelin (d18:0/12:0)	1.73	0.81	5.2×10^{-4}	8.7×10^{-3}	Down
35. Naphthol AS-BI ?-D-glucuronide	1.66	0.12	9.0×10^{-4}	0.011	Down
36. Ganglioside GM2 (d18:1/14:0)	1.64	0.66	9.4×10^{-4}	0.011	Down
37. Man-?1-6-Ins-1-P-Cer(t20:0/2-OH-26:0)	1.60	0.79	0.001	0.014	Down
38. 2-Hydroxy-4-(methylthio)butanoic acid	1.60	0.79	0.001	0.012	Down
39. Heptanoylcarnitine	1.58	0.68	0.002	0.018	Down
40. Stercobilin	1.44	0.07	0.003	0.021	Down
41.5-Amino-2-[(2-amino-3-carboxypropanoyl)oxyamino]pentanoic acid	1.41	0.72	0.007	0.034	Down
42.[4-(3,4-difluorophenyl)-1,1-dioxo-1$l^{6},4-benzothiazin-2-yl]-morpholin-4-ylmethanone	1.34	0.62	0.007	0.034	Down
43. Tetrachlorophthalic anhydride	1.30	0.80	0.007	0.034	Down
